# Smoking marijuana in public: the spatial and policy shift in New York City arrests, 1992–2003

**DOI:** 10.1186/1477-7517-3-22

**Published:** 2006-08-04

**Authors:** Andrew Golub, Bruce D Johnson, Eloise Dunlap

**Affiliations:** 1 Department of Sociology, University of Vermont, Burlington, VT, USA; 2 Institute for Special Populations Research, National Development and Research Institutes, Inc. New York, NY, USA

## Abstract

**Background:**

During the 1990s, the New York Police Department (NYPD) greatly expanded arrests for smoking marijuana in public view (MPV). By 2000, MPV accounted for 15% of all arrests. The NYPD's supporters report this arrest activity is just part of quality-of-life (QOL) policing, which seeks to promote order in public locations by aggressively patrolling for behaviors that offend the general population. The NYPD's critics contend the NYPD has disproportionately targeted poor, black and Hispanic communities.

**Methods:**

This paper analyzes the geographic distribution of MPV arrests from 1992 to 2003 to evaluate these alternative perspectives. A sequence of maps identify that the focus of MPV arrests shifted over time.

**Results:**

In the early 1990s, most MPV arrests were recorded in the lower half of Manhattan (NYC's business and cultural center) and by the transit police. However, in the later 1990s and into the 2000s, most MPV arrests were recorded in high poverty, minority communities outside the lower Manhattan area and by the NYPD's policing of low-income housing projects.

**Conclusion:**

These findings suggest that current levels of MPV arrests in NYC may not be justifiable, at least based solely on the purpose of QOL policing. Accordingly, we suggest the NYPD seriously consider less stringent measures for public marijuana smokers, especially for use outside of highly public locations in recessed locations hidden from open view (like the stairwell of a housing project). Alternatives could include Desk Appearance Tickets, fines, or simply requiring smokers to desist, discard their product, and move along.

## Background

During the 1990s, the New York City Police Department (NYPD) greatly expanded arrests for marijuana possession. King and Mauer noted that by the late 1990s that marijuana arrests (mostly for possession) constituted nearly half of all drug arrests nationwide [1]. Golub, Johnson and Dunlap confirmed that this was also the case in New York City [2]. Moreover, the vast majority of marijuana arrests (83%) were for criminal possession of marijuana in the fifth degree (NYS Penal Law 221.10), a Class B misdemeanor. New York State (NYS) specifies this charge pertains, " [w]hen he knowingly and unlawfully possesses marihuana in a public place...and **such marihuana is burning or open to public view**; or, ...weight of more than twenty-five grams [3]." We refer to this charge as MPV, which can alternatively stand for "marijuana in public view" or "marijuana possession, fifth (V) degree." This paper analyzes the spatial distribution of MPV arrests throughout New York City (NYC) from 1992 to 2003 to better understand the shifting focus of this arrest initiative, to examine the communities most impacted, and to identify major shifts in enforcement. In particular, this paper analyzes whether the geographic distribution of MPV arrests was more consistent with the goals of NYPD's policing initiatives or with the claims by the critics of this NYPD policy.

The use of arrest for controlling smoking marijuana in public has been included in the NYPD's focus on quality-of-life (QOL) policing. The QOL program seeks to maintain order in public spaces by aggressively enforcing laws with arrest against minor offenses that occur in public and that can be offensive to the general population. A variety of publications provide extensive descriptions of the QOL program, its implementation, and its evolution [4-8]. Johnson et al. provide a more focused history of marijuana policy and law enforcement in NYC [9]. A New York Times article appearing in 1998 describes how MPV arrests grew as part of QOL policing, "Arrests on marijuana charges have jumped to a record number this year, driven by the Giuliani administration's 'zero tolerance' approach [a near synonym for QOL policing] that has police officers pursuing anyone found possessing, selling or smoking even small amounts of marijuana [10]." Advocates of QOL policing have credited this policing effort with reclaiming public spaces, increasing tourism, and reducing both minor and more serious crimes, including murder [4-8]. This perspective informed our first hypothesis regarding the geographic distribution of MPV arrests.

**Hypothesis 1: **QOL policing has targeted public spaces for MPV arrests.

**Figure 1:**
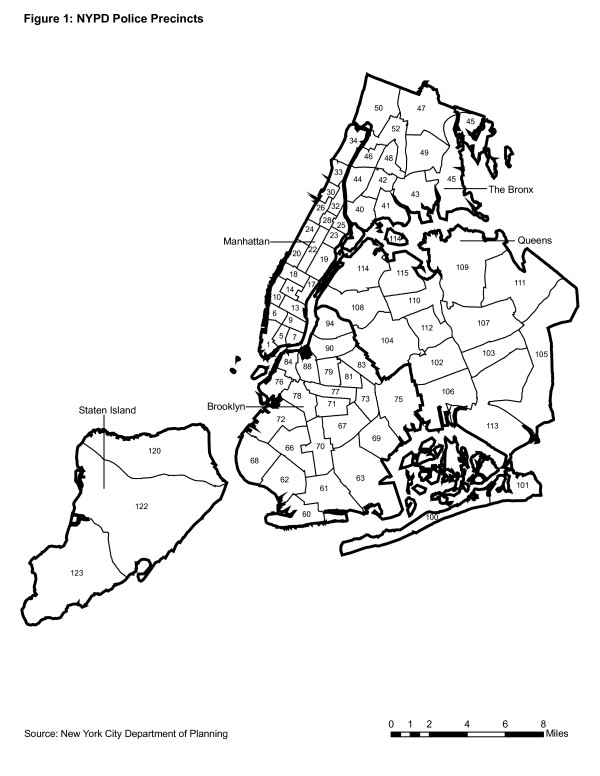


To the extent that MPV arrests have been consistent with Hypothesis 1, they should tend to have occurred in areas routinely frequented for leisure by residents and tourists. Most NYC neighborhoods have many streets and public spaces such as parks that serve this purpose. Accordingly, Hypothesis 1 suggests that MPV arrests should occur throughout NYC. However, the showcase of NYC's business center, cultural offerings, and most popular leisure destination has been lower Manhattan (generally below 110^th ^Street) including Central Park, the Theatre District, the midtown business district, skyscrapers, museums, and walking areas with shops, restaurants, cafés and nightclubs including Greenwich Village and Chinatown, among others. Figure 1 provides a map of NYC indicating the location of NYPD precincts. Table 1 provides a list of the approximate neighborhoods served by each precinct. The NYPD also maintains seven specialized precincts (see Table 1) that are not organized by specific neighborhood including Transit and Housing. NYPD's transit police provide safety and enforce numerous criminal and civic order codes on NYC's widely-used public subway and bus services. The Housing police patrol numerous public housing projects located throughout the city, but mainly in low-income neighborhoods. According to Hypothesis 1, the lower Manhattan and transit precincts should record a concentration of enforcement for QOL violations, including MPV arrests, to the extent that individuals tend to smoke marijuana in those locations.

**Table 1:** An Approximate Mapping of NYPD Precincts to NYC Community Districts

**NYPD Prec.**	**Comm.District**	**Neighborhoods**
		***Manhattan***
1	1	Battery Park, Tribeca
5	3	Lower East Side, Chinatown
6	2	Greenwich Village, Soho
7	3	Lower East Side, Chinatown
9	3	Lower East Side, Chinatown
10	4	Chelsea, Clinton
13	6	Stuyvesant Town, Turtle Bay
14	5	Midtown Business District
17	6	Stuyvesant Town, Turtle Bay
18	4	Chelsea, Clinton
19	8	Upper East Side
20	7	West Side, Upper West Side
22		Central Park
23	11	East Harlem
24	7	West Side, Upper West Side
25	11	East Harlem
26	9	Manhattanville, Hamilton Heights
28	10	Central Harlem
30	9	Manhattanville, Hamilton Heights
32	10	Central Harlem
33	12	Washington Heights, Inwood
34	12	Washington Heights, Inwood
		
		***The Bronx***
40	1	Melrose, Mott Haven, Port Morris
41	2	Hunts Point, Longwood
42	3	Morrisania, Crotona Park East
43	9	Soundview, Parkchester
44	4	Highbridge, Concourse Village
45	10	Throgs Neck, Co-op City, Pelham Bay
46	5	University Heights, Fordham, Mt. Hope
47	12	Wakefield, Williamsbridge
48	6	East Tremont, Belmont
49	11	Pelham Parkway, Morris Park, Laconia
50	8	Riverdale, Kingsbridge, Marble Hill
52	7	Bedford Park, Norwood, Fordham
		
		***Brooklyn***
60	13	Coney Island, Brighton Beach
61	15	Sheepshead Bay, Gerritsen Beach
62	11	Bensonhurst, Bath Beach
63	18	Canarsie, Flatlands
66	12	Borough Park, Ocean Parkway
67	17	East Flatbush, Rugby, Farragut
68	10	Bay Ridge, Dyker Heights
69	18	Canarsie, Flatlands
70	14	Flatbush, Midwood
71	9	Crown Heights South, Wingate
72	7	Sunset Park, Windsor Terrace
73	16	Brownsville, Ocean Hill
75	5	East New York, Starrett City
76	6	Park Slope, Carroll Gardens
77	8	Crown Heights North
78	6	Park Slope, Carroll Gardens
79	3	Bedford Stuyvesant
81	3	Bedford Stuyvesant
83	4	Bushwick
84	2	Brooklyn Heights, Fort Greene
88	2	Brooklyn Heights, Fort Greene
90	1	Williamsburg, Greenpoint
94	1	Williamsburg, Greenpoint
		
		***Queens***
100	14	The Rockaways, Broad Channel
101	14	The Rockaways, Broad Channel
102	9	Woodhaven, Richmond Hill
103	12	Jamaica, St. Albans, Hollis
104	5	Ridgewood, Glendale, Maspeth
105	13	Queens Village, Rosedale
106	10	Ozone Park, Howard Beach
107	8	Fresh Meadows, Briarwood
108	2	Sunnyside, Woodside
109	7	Flushing, Bay Terrace
110	4	Elmhurst, South Corona
111	11	Bayside, Douglaston, Little Neck
112	6	Forest Hills, Rego Park
113	12	Jamaica, St. Albans, Hollis
114	1	Astoria, Long Island City
115	3	Jackson heights, North Corona
		
		***Staten Island***
120	1	Stapleton, Port Richmond
122	2	New Springville, South Beach
123	3	Tottenville, Woodrow, Great Kills
		
		***Other Precincts***
		NYC Transit
		NYC Housing
		Park Police
		NYPD Headquarters
		Triboro Bridge/Tunnel
		Port Authority of NY/NJ
		Metro Transit

Other scholars have questioned whether the improvements observed in NYC, particularly the reduction in crime, resulted from policing initiatives or from other factors such as the decline of the crack epidemic and its violent drug markets [11-15]. During the 1990s, marijuana supplanted crack as the drug-of-choice among youths, especially in the inner city [16,17]. Moreover, some critics have charged that the NYPD's aggressive law enforcement efforts target ethnic minorities and the poor [18-20]. Golub, Johnson and Dunlap has already established that most MPV arrestees in each year from 1980 to 2003 were either black or Hispanic (percentages in each year ranged from 74% to 91%) [2]. However, that analysis did not consider where arrests occurred. This paper examines variation in MPV arrests across precincts and over time. This alternative perspective led to our second hypothesis.

**Hypothesis 2: **MPV arrests have targeted persons primarily in poor, black and Hispanic communities.

To the extent that MPV arrests have been consistent with Hypothesis 2, they should tend to have been recorded by those precincts throughout NYC's five boroughs with the highest percentages of black, Hispanic and poor residents, and by the housing police.

## Methods

This paper examines a series of 12 maps showing MPV arrests by precinct from 1992 to 2003 and describes the extent that the distribution of arrests in each year is most consistent with either of the two hypotheses presented above. This section describes the analytic procedures and the two data sources employed: NYS official records of arrests and the Decennial Census. Hypothesis 1 predicts that MPV arrests would be recorded citywide, but with a concentration in lower Manhattan and by transit. Lower Manhattan was operationalized as those areas served by precincts 1 through 24, an area extending from the southern tip of Manhattan up through Central Park and including the upper East and West side neighborhoods on either side of the park (see Figure 1). This lower Manhattan territory excludes the predominately black and Hispanic neighborhoods in northern Manhattan such as Harlem and Washington Heights and excludes the four outer boroughs of NYC.

**Figure 2:**
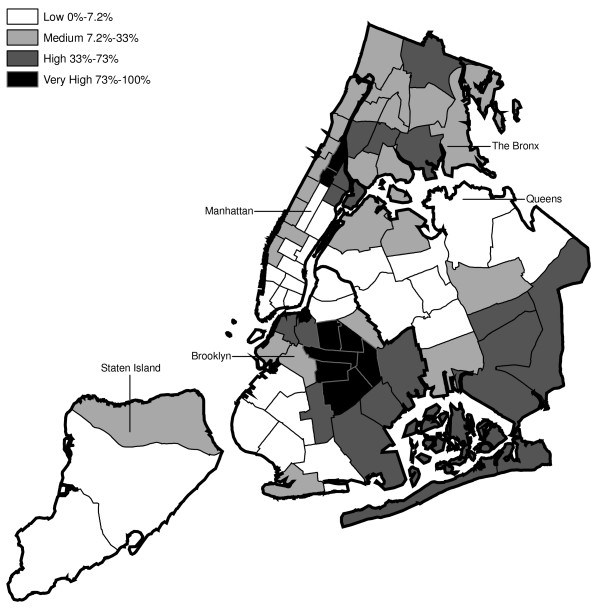
Distribution of Black Residents across NYPD Precincts (2000 Census Data).

Hypothesis 2 predicts that MPV arrests would tend to be recorded by those precincts serving low-income ethnic minority communities and by the housing police. Figures 2 through 4 present the estimated percentage of residents by precinct that are black, Hispanic and have income below poverty level, respectively, based on the 2000 Census data. Each figure classifies the precincts into thirds (low, medium and high) according to the demographic characteristic analyzed. The upper third is further divided into the top tenth (very high) and the 10^th ^to 33^rd ^percentile (high).

**Figure 3:**
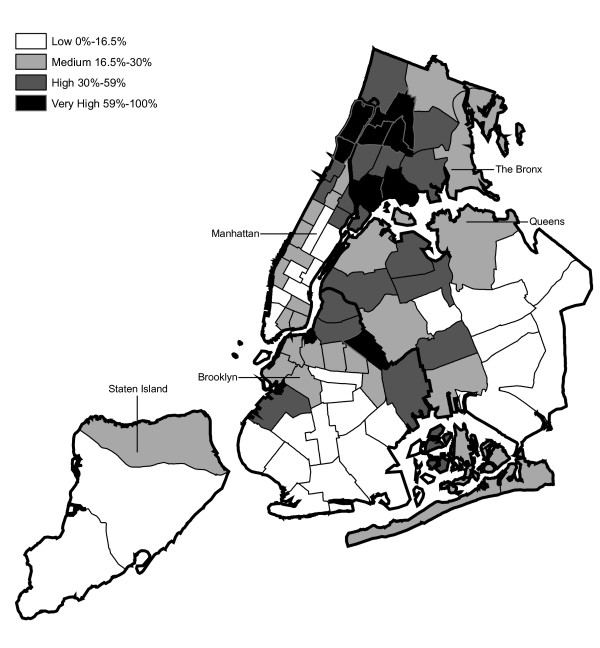
Distribution of Hispanic Residents across NYPD Precincts (2000 Census Data).

**Figure 4:**
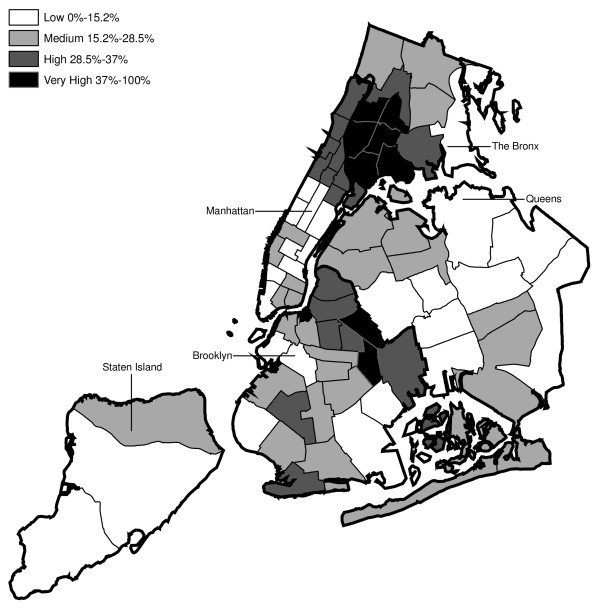
Distribution of Impoverished Residents across NYPD Precincts (2000 Census Data).

Two graphs examine the distribution of MPV arrests across the 1992–2003 study period as predicted by each hypothesis to ascertain the extent to which each pertains over time. Regarding Hypothesis 1, a graph examines the percentage of MPV arrests in each year occurring in lower Manhattan as opposed to other neighborhood precincts. The graph also indicates the percentage of arrests citywide recorded by transit and by housing. The other five specialized precincts were combined and displayed as cases not included in the other four categories. The number of MPV arrests in those five specialized precincts never exceeded 2% in any given year.

Regarding Hypothesis 2, a graph examines the percentage of MPV arrests in each year recorded in precincts that are in the top third by race/ethnicity group and poverty status (high or very high in Figures 2, 3, 4). This second graph excludes arrests occurring in the seven specialized precincts not associated with a specific neighborhood.

### NYS arrests

The Division of Criminal Justice Services (DCJS) maintains the official arrest records for New York State. These records are routinely used to produce criminal histories (rap sheets) in support of law enforcement activities. Under special arrangement, the project obtained records of all adult arrests recorded in NYC from January 1, 1980 through January 12, 2005. All personal identifiers (names, addresses, criminal IDs) were removed from the research dataset. Each record contains a date, an arrest charge and demographic information. Race/ethnicity is coded as either white, black, Hispanic, other or missing.

This dataset also includes "sealed" arrests. For many minor arrests, the judge will issue an "adjudication in contemplation of dismissal." If the arrestee is not rearrested within a period of time (often the next six months), the state will seal the record of an arrest. Sealed arrests are not provided on rap sheets generated for subsequent arrests. Golub, Johnson and Dunlap document that over 85% of MPV arrests 1992–2003 were sealed [2]. Consequently, exclusion of sealed arrests from this analysis would have led to a serious undercount of aggregate MPV arrest activity.

This study examined MPV arrests occurring in NYC up to December 30, 2003, to assure that the dataset was virtually complete over the period of analysis. NYS law enforcement agencies are sometimes delayed sending their reports to DCJS. Steven Greenstein of DCJS estimated that the data for 2003 should be more than 98% complete [21]. The complete 1980–2003 dataset includes records of 305,506 adult MPV arrests recorded in NYC.

### Decennial census

Every ten years, the Decennial Census counts the population of U.S. residents [22]. This analysis used the census data to identify the areas of NYC with the highest concentrations of minorities and poverty. This section describes limitations to the census data and to their use in this analysis. A primary limitation is that the census provides a single snapshot of the population. These data are for 2000 and do not account for shifts in the NYC population occurring 1992–2003. Additionally, the U.S. Census Bureau reported that the program tends to undercount the population especially blacks, Hispanics, and persons of lower SES [23]. However, the distribution of race/ethnicity and poverty across precincts (see Figures 2, 3, 4) generally accorded with the authors' understanding of NYC confirming that the census 2000 data were sufficiently accurate for the purposes of this study.

The census data for this study were available online in nearly the exact format needed on a website maintained by the NYC Department of City Planning [24]. The only difference was that the census data were aggregated according to Community Districts instead of precincts. However, NYC's Community Districts have very similar boundaries as NYPD precincts. Each NYPD precinct was matched to its corresponding or most closely matching community district (see Table 1). The correspondence between community districts and precincts is best outside of Manhattan. In the Bronx, there is an exact one-to-one correspondence between them; only the numbering differs (see Table 1). In Staten Island, the boundaries separating the two southern aggregations differ slightly. In Queens, two community districts span two NYPD precincts each. When more than one precinct is contained within a community district, the census data for the whole community district were linked with each precinct. In Brooklyn, five community districts span two NYPD precincts each. In Manhattan above 57^th ^Street, five of the six community districts contain two precincts. Below 57^th ^Street, there are 10 precincts to six community districts and the borders between them do not match up. For Manhattan, precincts were matched with the community district covering the largest portion of it.

Another difficulty is that the census uses different race/ethnicity categories than the NYS arrest data. The census asks separate questions about Hispanic/Latino origin and race [22]. Respondents designate one or more racial categories including white, black/African American/Negro, and 13 others. For this study, census respondents that reported they were white, non-Hispanic, and not of mixed race were classified as white. Respondents that reported they were black, non-Hispanic, and not of mixed race were classified as black. The project used the older designation black for consistency with the NYS arrest designation and because the term is more inclusive than African American; many black New Yorkers consider themselves to be of Caribbean as opposed to African origin and others do not have U.S. citizenship. Respondents that reported Hispanic origin were designated as Hispanic, regardless of their designated race.

## Findings

**Figure 5:**
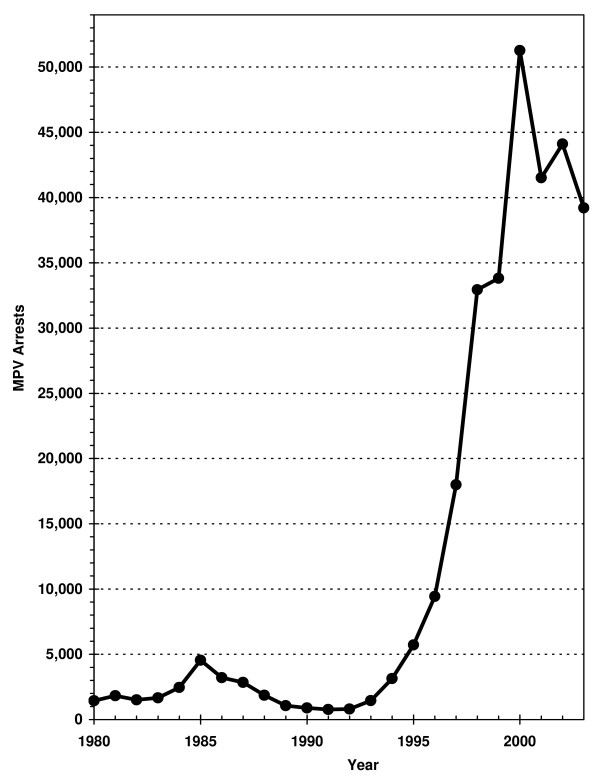
Adult MPV Arrests in NYC, 1980–2003.

During the 1980s, MPV arrests in NYC rose from 1,400 (all counts have been rounded to the nearest hundred) in 1980 up to a peak of 4,500 in 1985 and then declined to a new low of 800 in 1991 (see Figure 5). During the 1990s, MPV arrests increased slowly and then more rapidly leading to a peak of 51,000 (2000) at which time it became the most common misdemeanor arrest charge in NYC [2].

**Figure 6:**
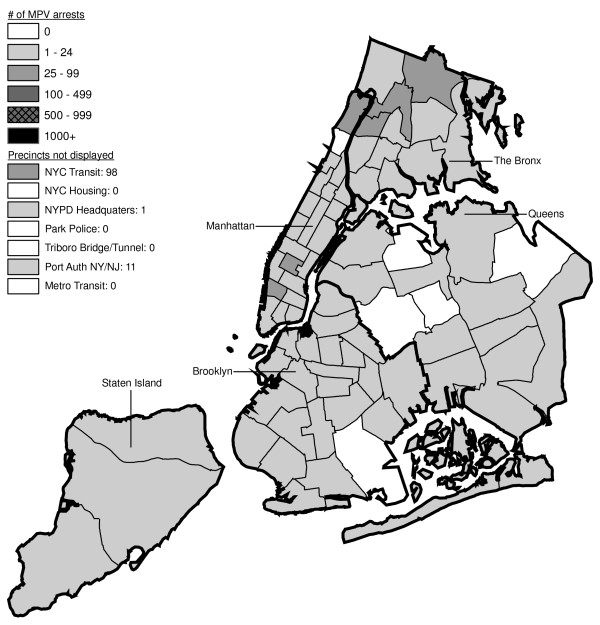
Marijuana in Public View Arrests by NYPD Precinct in 1992 (n = 672).

Figures 6 through 17 present the geographic distribution of MPV arrests throughout NYC from 1992 to 2003, respectively. (A small proportion of the MPV arrests (2%) recorded in the dataset did not indicate the precinct responsible for the arrest. These arrests were excluded from Figures 6 through 17. Accordingly, the number of cases by year as indicated in these figures is less than the total number of arrests identified in Figure 5.)

**Figure 7:**
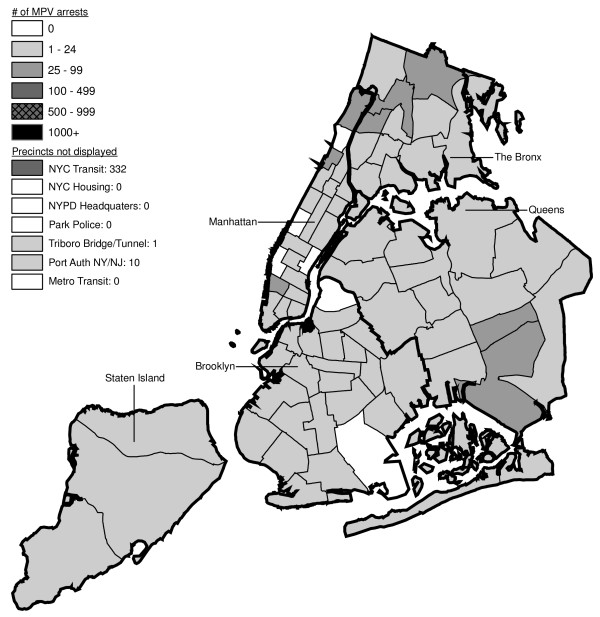
Marijuana in Public View Arrests by NYPD Precinct in 1993 (n = 1,082).

**Figure 8:**
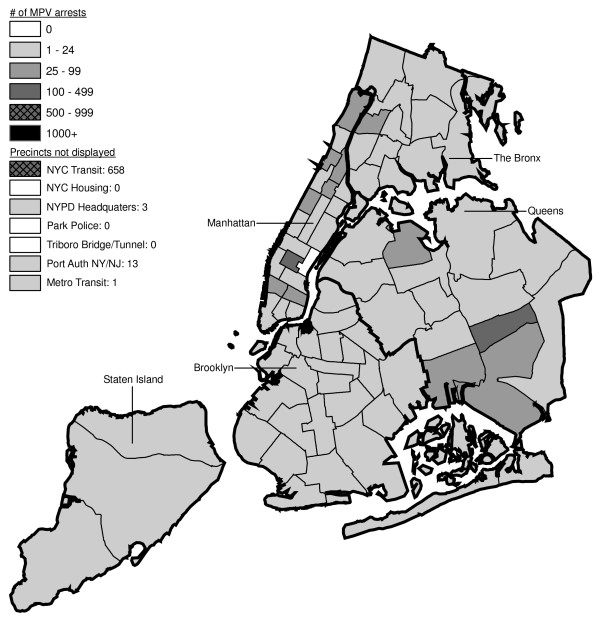
Marijuana in Public View Arrests by NYPD Precinct in 1994 (n = 1,851).

**Figure 9:**
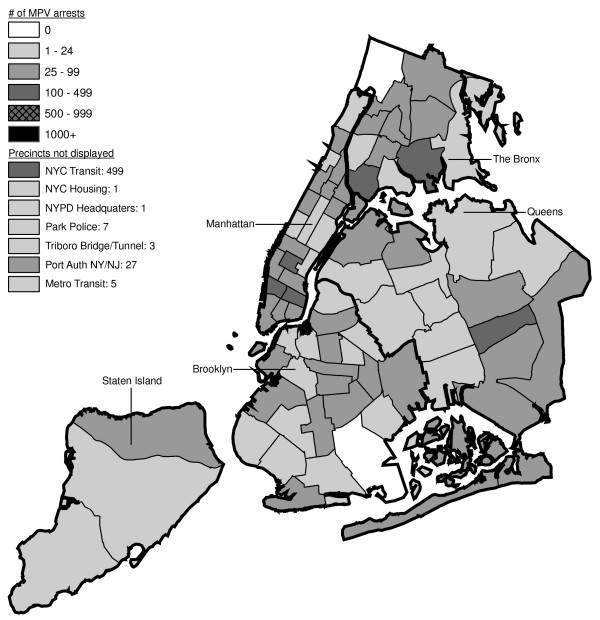
Marijuana in Public View Arrests by NYPD Precinct in 1995 (n = 3,973).

**Figure 10:**
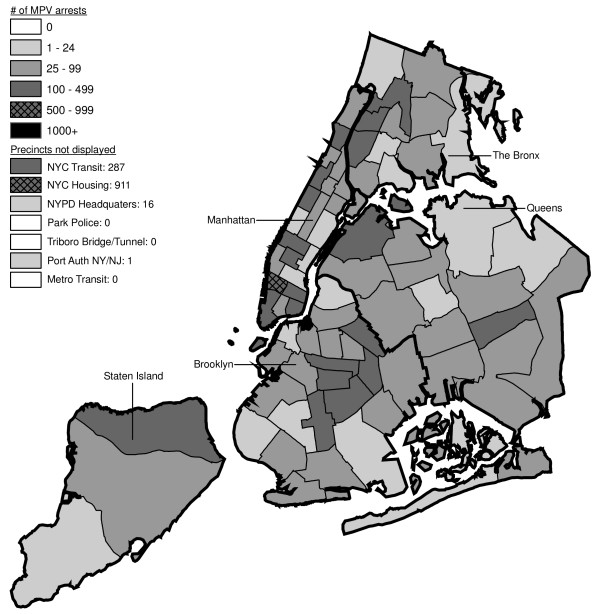
Marijuana in Public View Arrests by NYPD Precinct in 1996 (n = 8,377).

**Figure 11:**
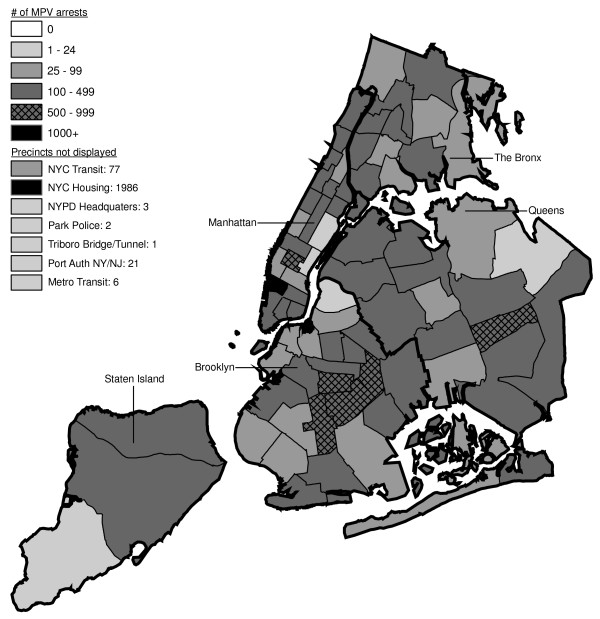
Marijuana in Public View Arrests by NYPD Precinct in 1997 (n = 17,794).

**Figure 12:**
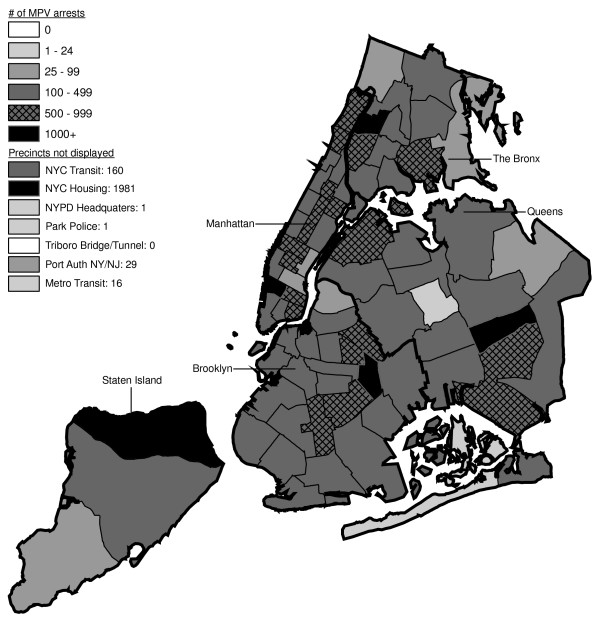
Marijuana in Public View Arrests by NYPD Precinct in 1998 (n = 32,937).

**Figure 13:**
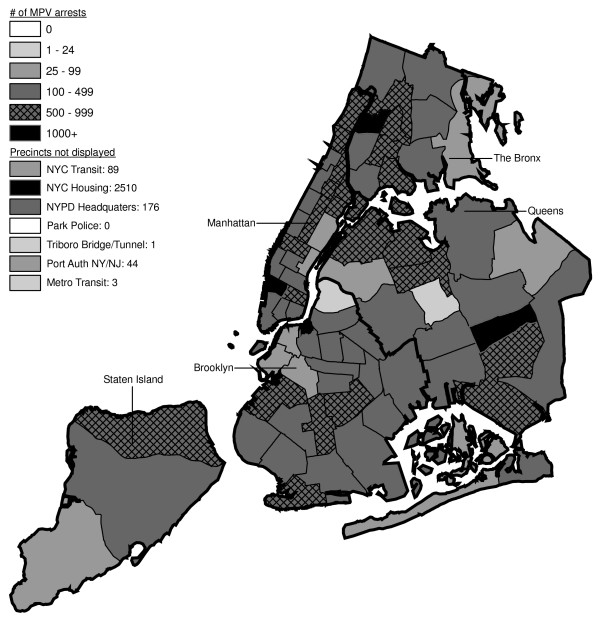
Marijuana in Public View Arrests by NYPD Precinct in 1999 (n = 33,817).

**Figure 14:**
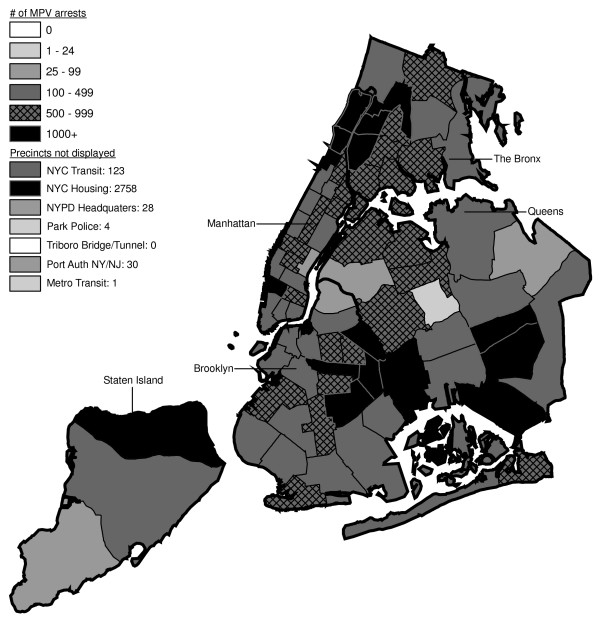
Marijuana in Public View Arrests by NYPD Precinct in 2000 (n = 51,267).

**Figure 15:**
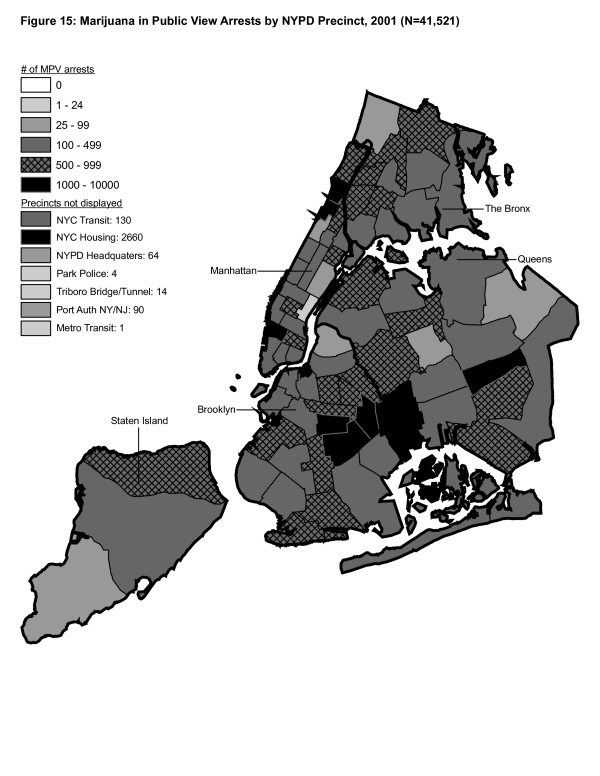
Marijuana in Public View Arrests by NYPD Precinct in 2001 (n = 41,521).

**Figure 16:**
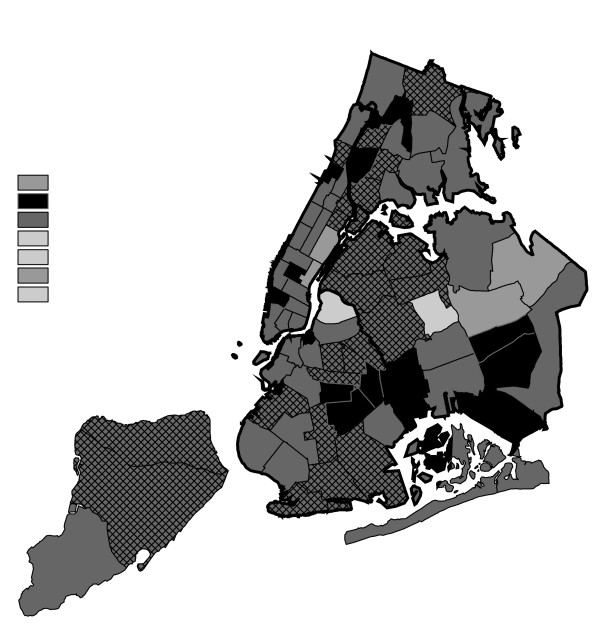
Marijuana in Public View Arrests by NYPD Precinct in 2002 (n = 44,110).

**Figure 17:**
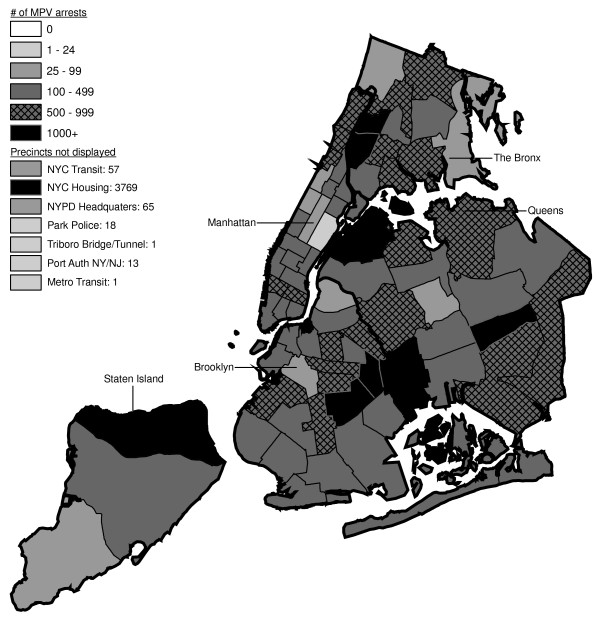
Marijuana in Public View Arrests by NYPD Precinct in 2003 (n = 39,212).

The distribution of MPV arrests in 1992 (Figure 6) is mostly consistent with Hypothesis1. MPV arrests were scattered broadly throughout the city; most precincts reported 1 to 24 MPV arrests. There were several areas of concentration in MPV arrests at this initial point: a) Greenwich Village and Soho, a popular area among tourists in downtown Manhattan; b) transit; and c) a swath through northern Manhattan (Washington Heights) leading into three precincts in central Bronx, poor Hispanic areas except for the northernmost part of the Bronx which is predominately black and less impoverished (see Figures 2, 3, 4). Only the concentration in the northern part of the city provides any suggestion that NYPD may have targeted some (but not all) poor ethnic-minority communities.

From 1992 through 1996 (Figures 6, 7, 8, 9), transit was a major enforcer of MPV arrests. In 1994 (Figure 8), transit recorded more than a third (36%) of all MPV arrests. As of 1995 (Figure 9), transit was still the largest enforcer of the MPV arrest policy. However, starting in 1995, the number of MPV arrests recorded by transit started a precipitous decline. Several new concentrations of MPV arrests emerged. Consistent with Hypothesis 1, several sections of downtown (Greenwich Village, Soho, and the Lower East Side, Chinatown) as well as the midtown business district were primary, recording more than 100 MPV arrests in 1995. However, consistent with Hypothesis 2, a poor Hispanic section of the Bronx (Melrose, Mott Haven, Port Morris), a poor mixed black-Hispanic section of the Bronx (Soundview, Parkchester), and a relatively wealthier black section of Queens (Jamaica, St. Albans, Hollis) also had more than 100 MPV arrests.

A major shift in MPV enforcement started in 1996 (Figure 10). Starting that year, housing emerged as the largest enforcer of MPV arrests, with more than three times as many MPV arrests as transit. In 1996, there were numerous precincts recording more than 100 arrests. Consistent with Hypothesis 1, many of them were in Manhattan, especially Downtown and Midtown (precincts below Central Park). However, consistent with Hypothesis 2, a new concentration of MPV arrests emerged in the middle of Brooklyn. All of these precincts are heavily black except for Bushwick, which is predominately Hispanic. Two of the precincts (Bushwick and Brownsville, Ocean Hill) were particularly impoverished; the other four were relatively wealthier.

From 1996 to 2000, MPV arrests grew in various ways consistent with both Hypothesis 1 and 2. In 2000, there were heavy concentrations of 500 or more MPV arrests each in several midtown precincts, Central Park, and downtown precincts (Greenwich Village, Soho had over 1,000) (Figure 14). However consistent with Hypothesis 2, 15 precincts in northern Manhattan and the outer boroughs registered 1,000 or more MPV arrests. Also consistent with Hypothesis 2, housing recorded the most MPV arrests (2,758) in 2000, a figure that increased to 3,637 in 2002 and to 3769 in 2003 (Figures 16, 17). Central Park is the largest open space in Manhattan; the number of MPV arrests rose to over 500 per year (1998–2000, Figures 12, 13, 14), but declined to 100–499 (2001–2002, Figures 15, 16), and to under 100 in 2003 (Figure 17).

By 2003, evidence of MPV as part of QOL policing had become more limited (Figure 17). Not one precinct in Manhattan registered 1,000 or more MPV arrests. In contrast, eight precincts in the outer boroughs had 1,000 or more; this included two poor Hispanic sections of the Bronx (Highbridge, Concourse Village and University Heights, Fordham, Mt. Hope), two poor black and Hispanic sections of Brooklyn (Brownsville, Ocean City and East New York, Starrett City), a wealthier black section of Brooklyn (East Flatbush, Rugby, Farragut), a wealthier black section of Queens (Jamaica, St. Albans, Hollis), and two very mixed wealthier communities in Queens (Astoria, Long Island City) and Staten Island (Stapleton, Port Richmond). Housing recorded 3,769 MPV arrests accounting for 10% of the total in 2003.

**Figure 18:**
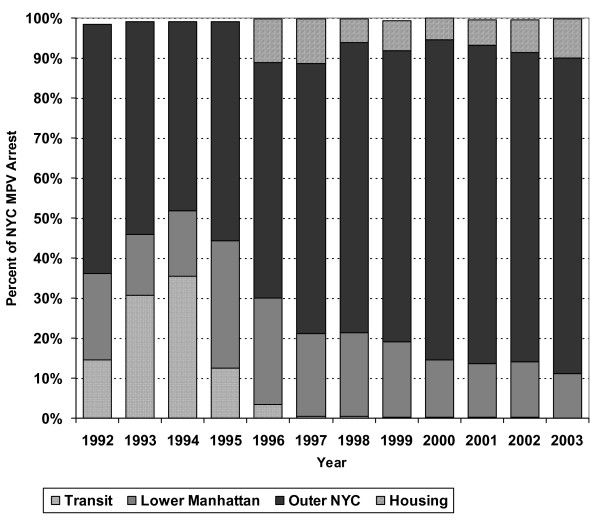
Adult MPV Arrests in Lower Manhattan (up through Central Park) as a Percent of All New York City MPV Arrests, 1992–2003.

Figure 18 identifies the percentage of MPV arrests in lower Manhattan. Consistent with Hypothesis 1, in 1992 more than a third of all MPV arrests were recorded in lower Manhattan or by transit. This combined percentage rose to more than 50% in 1994. However, this percentage started a steady decline in 1995 dropping to just over 10% by 2003. These findings suggest that MPV arrests became less focused on QOL policing in highly public locations of the downtown Manhattan business and entertainment district over time and shifted to the outer boroughs.

**Figure 19:**
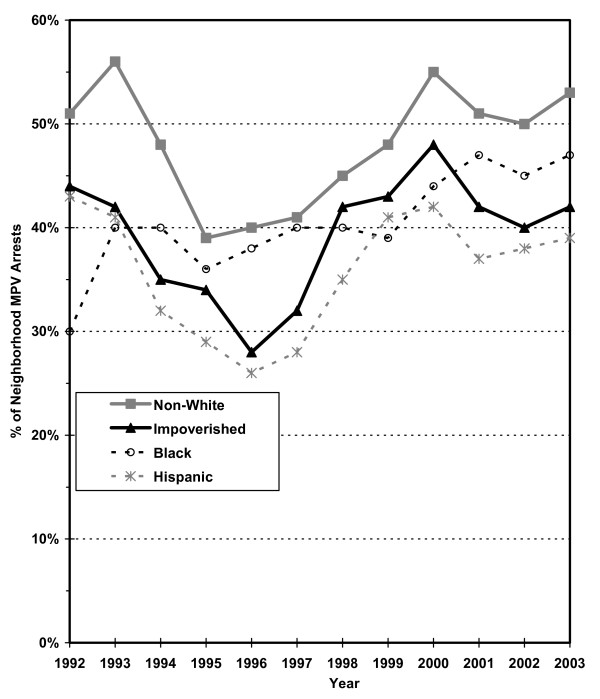
Percent of Adult MPV Arrests in the Most Non-White, Impoverished, Black and Hispanic Areas (Upper Third) of New York City, 1992–2003 (analysis limited to neighborhood precincts).

Figure 19 identifies the percentage of MPV arrests occurring in precincts with high concentrations of non-white and impoverished persons. As of 1992, more than half the MPV arrests occurred in precincts with high percentages of non-whites and nearly half (45%) where in high poverty areas. The non-white percentage declined to just under 40% by 1995 and the high poverty percentage declined to less than 30% by 1996. However, after 1996, each MPV arrest index increased back to peak levels by 2000 of more than 50% non-white and just under 50% high poverty, where they approximately remained through 2003. These findings indicate that a very substantial proportion of MPV arrests occurred in high poverty black and Hispanic communities and that this targeting increased moderately from a low in 1995–1996 up to 2000 – and has remained at a slightly lower level in 2001–03. Combined with the findings of Figure 18 and a broad reading of the maps, these findings suggest that much MPV arrest activity declined in highly public locations over time extending to the outer boroughs with an emphasis in high poverty black and Hispanic communities.

## Conclusion

This analysis indicates that the geography and perhaps even the purpose of MPV arrests in NYC shifted over time. In the early 1990s, from a third to a half of all MPV arrests were recorded in highly-public sections of Manhattan or in NYC's transit service. This pattern accords with the focus of QOL policing, on maintaining order by enforcing laws against offensive behaviors in public locations. Moreover, it stands in contrast to critics' claims that the NYPD targeted poor ethnic minority communities for MPV arrests. On the other hand, the critics concerns are supported by the fact that most MPV arrestees during the early 1990s were black or Hispanic, even though the arrests took place in areas that were not predominately black or Hispanic [25].

The critics' concerns are further supported by changes over time. Starting in 1995 and continuing into the 2000s, MPV arrests shifted to less public locations. By 2003, MPV arrests were recorded primarily outside of Manhattan and in poorer neighborhoods with higher concentrations of blacks and Hispanics. This transformation raises questions about the purpose of the vast number of MPV arrests in NYC into the 2000s. These MPV arrests do not appear to primarily serve the goals of QOL policing. Moreover, controlling marijuana use in NYC hardly advances other traditional goals of crime control. During the crack epidemic, drug binges and open-air drug markets were associated with extensive violence. Thus, controlling crack and its associated activities could be justified as attacking the roots of much of the prevailing crime and violence. The current situation with marijuana is much less critical. Marijuana use and its sales in NYC have been associated much less with other forms of criminal activity.

Recent articles in the New York Times indicate that as of 2006 Mayor Bloomberg planned to continue QOL policing [26]. The findings of this study suggest that Mayor Bloomberg and the NYPD should carefully re-examine the current practices regarding arrests for smoking marijuana in public. It appears that the current MPV arrest practices have been unnecessarily exacerbating race relationships, and contributing substantially to racial/ethnic disparities in arrest and dispositions [2].

To some extent, this MPV arrest policy, based on restoring order in highly-public locations may have been quite successful in transit, Central Park, and lower Manhattan, so that fewer MPV arrests now occur where many had occurred in the late 1990s. It is far less clear that the numerous MPV arrests occurring in low-income neighborhoods in the outer boroughs have succeeded at preventing marijuana smoking in public locations. Less stringent alternatives to arrest would likely meet the city's goals of maintaining order. The NYPD may develop special procedures for dealing with marijuana use in recessed locations that are well hidden from open view, but are nonetheless public spaces such as the stairwells of housing projects or a hidden section of a park. Alternatives to MPV arrests and detention could include Desk Appearance Tickets (DATs), C-Summons (which require a court appearance like a DAT but for a violation as opposed to a non-criminal offense), or simply requiring smokers to desist, discard their product, and move along (also see [9]).
